# Proteomic Analysis Identifies p62/SQSTM1 as a Critical Player in PARP Inhibitor Resistance

**DOI:** 10.3389/fonc.2022.908603

**Published:** 2022-06-29

**Authors:** Mohammed Hafiz Uddin, Jun-Ying Zhou, Julio Pimentel, Steve M. Patrick, Seongho Kim, Malathy P. Shekhar, Gen Sheng Wu

**Affiliations:** ^1^ Molecular Therapeutics Program, Karmanos Cancer Institute, Wayne State University School of Medicine, Detroit, MI, United States; ^2^ Cancer Biology Program, Wayne State University School of Medicine, Detroit, MI, United States; ^3^ Department of Oncology, Wayne State University School of Medicine, Detroit, MI, United States

**Keywords:** PARP inhibitor, resistance, TNBC, p62/SQSTM1, proteomics, autophagy, rapamycin

## Abstract

Poly (ADP-ribose) polymerase (PARP) inhibitors (PARPis) are currently being used for treating breast cancer patients with deleterious or suspected deleterious germline BRCA-mutated, HER2-negative locally advanced or metastatic diseases. Despite durable responses, almost all patients receiving PARPis ultimately develop resistance and succumb to their illness, but the mechanism of PARPi resistance is not fully understood. To better understand the mechanism of PARPi resistance, we established two olaparib-resistant SUM159 and MDA468 cells by chronically exposing olaparib-sensitive SUM159 and MDA468 cells to olaparib. Olaparib-resistant SUM159 and MDA468 cells displayed 5-fold and 7-fold more resistance over their corresponding counterparts. Despite defects in PARPi-induced DNA damage, these olaparib-resistant cells are sensitive to cisplatin-induced cell death. Using an unbiased proteomic approach, we identified 6 447 proteins, of which 107 proteins were differentially expressed between olaparib-sensitive and -resistant cells. Ingenuity pathway analysis (IPA) revealed a number of pathways that are significantly altered, including mTOR and ubiquitin pathways. Among these differentially expressed proteins, p62/SQSTM1 (thereafter p62), a scaffold protein, plays a critical role in binding to and delivering the ubiquitinated proteins to the autophagosome membrane for autophagic degradation, was significantly downregulated in olaparib-resistant cells. We found that autophagy inducers rapamycin and everolimus synergistically sensitize olaparib-resistant cells to olaparib. Moreover, p62 protein expression was correlated with better overall survival in estrogen receptor-negative breast cancer. Thus, these findings suggest that PARPi-sensitive TNBC cells hyperactivate autophagy as they develop acquired resistance and that pharmacological stimulation of excessive autophagy could lead to cell death and thus overcome PARPi resistance.

## Introduction

Triple-negative breast cancer (TNBC) accounts for ~15-20% of all breast cancer cases and has poor prognoses (1, 2). TNBC is clinically defined as lacking estrogen receptor and progesterone receptor expression and HER2 amplification (3, 4). Consequently, this aggressive disease does not respond to widely used targeted or endocrine therapies (5, 6). Patients with TNBC are treated with conventional chemotherapeutic agents (3), with an initial response to these chemotherapeutic agents, but relapse is inevitable (5). Therefore, there is a clinical need to develop a more effective treatment regimen for TNBC patients.

Despite lacking targeted therapies for the majority of patients with TNBC, ~70% of breast cancer patients carrying a germline BRCA1 mutation are triple-negative, and the incidence of BRCA mutations in TNBC varies from 16-42% (1, 7). One characteristic of BRCA-related cancers is that those tumors are homologous recombination (HR) repair defective. Because poly (ADP-ribose) polymerase (PARP) detects single-strand DNA breaks (SSB) and senses the enzymatic machinery to trigger base excision repair (BER), when the latter is inhibited by trapped PARP or PARP catalytic inhibition of a required repair process, damaged DNA is repaired through alternative pathways such as HR (8). Thus, targeting the BER pathway with PARP inhibitors (PARPis) in BRCA1/2 mutant cells disrupts both DNA repair pathways to induce tumor cell synthetic lethality. Based on this synthetic lethality interaction, a number of PARPis have been developed and approved for several cancers (9, 10). PARPis are effective against tumors with HR defects, including BRCA-related and BRCA-unrelated tumors (11, 12). For patients with breast cancer, PARPis are only used to treat patients with deleterious or suspected deleterious germline BRCA-mutated, HER2-negative locally advanced or metastatic breast cancer. Despite durable responses, almost all patients receiving PARPis eventually relapse and die from their metastatic disease. Several mechanisms of PARPi resistance have been proposed, including restoration of the BRCA gene by the acquisition of secondary mutations and increased drug efflux. However, the mechanisms of PARPi resistance are not fully understood.

p62/SQSTM1 (thereafter p62) was identified as the first protein that interacts with microtubule-associated protein 1A/1B-light chain 3 (LC3) to promote autophagy (13, 14). Autophagy is a highly conserved process by which cellular components are sequestered in double-membrane vesicles called autophagosomes (15). Upon fusion of autophagosomes with lysosomes, the cargo of the autophagosomes is degraded by lysosomal enzymes (16). In this process, p62 selectively interacts with LC3 and ubiquitinated proteins to initiate autophagosome formation and subsequent autophagy induction. p62 can also be an autophagy substrate, engulfed by autophagosomes and degraded by autophagolysosomes. p62 levels usually inversely correlate with autophagic degradation. Therefore, p62 levels are the standard of autophagic flux; low p62 levels indicate active autophagy, and high levels of p62 indicate low levels of autophagy.

In this study, we employed an unbiased proteomic approach to identify proteins and pathways that confer olaparib resistance. This approach led us to uncover several critical pathways associated with PARPi resistance in TNBC cells. Among these pathways, we showed that loss of p62 *via* autophagy plays a critical role in conferring olaparib resistance. We also found that the pharmacological induction of excessive autophagy effectively inhibits the growth of acquired olaparib-resistant TNBC cells. Thus, our study suggests that autophagy induction may be a viable strategy to overcome PARPi resistance.

## Materials and Methods

### Cell Culture

The TNBC cell lines SUM159 and MDA468 were obtained from the American Type Culture Collection (ATCC) (Rockville, MD, USA). Cells were grown in Dulbecco’s modified Eagle’s medium (DMEM) supplemented with 10% fetal bovine serum (FBS) and 1% penicillin and streptomycin. Cells were maintained at 37°C in a humidified atmosphere of 5% CO_2_ (CO_2_ water jacketed incubator (Series II), Forma Scientific Inc., Marzetta, OH, USA).

### Reagents and Antibodies

FBS (F0926), thiazolyl blue tetrazolium bromide (MTT) (M2128), cisplatin (232120), PVDF membranes (IPVH00010), and actin antibody (A1978) were purchased from Sigma-Aldrich (St. Louis. MO, USA). Trypsin-EDTA (25300–054), DMEM (11995-065), bovine serum albumin (BP1605-100), Col17A1 (MA5-24848), goat anti-mouse Alexa fluor 680 IgG (A21058), goat anti-rabbit Alexa fluor 680 IgG (A21109), and Lipofectamine 2000 (11-668-019) were purchased from ThermoFisher Scientific (Waltham, MA, USA). Penicillin-streptomycin (SV30010) was purchased from GE Healthcare (Chicago, IL, USA). The PARP inhibitor olaparib (O-9201) was obtained from LC Laboratories (Woburn, MA, USA). The mTOR inhibitor rapamycin (13346), everolimus (11597), and chloroquine (30708) were obtained from Cayman Chemicals (Ann Arbor, MI, USA). The protein assay dye (500-0006) was purchased from Bio-Rad (Hercules, CA, USA). RIPA Buffer (9806), S100A9 (72590), p62 (5114), GAPDH (5174), and LC3 (2775) antibodies were purchased from Cell Signaling Technologies (Danvers, MA, USA).

### Establishment of Olaparib-Resistant Cell Lines

Olaparib-resistant SUM159 (SUM159-R) and MDA468 (MDA468-R) cell lines were established by exposing parental SUM159 and MDA468 cells to increasing concentrations of olaparib over six months. Resistant cells (a pool of cells) were maintained in 25 µM of olaparib. Both parental and resistant SUM159 and MDA468 cells were authenticated through the genotyping service in the Karmanos Cancer Institute Biobanking Core.

### Knockdown of p62 by Short RNA Interfering RNA (siRNA)

siGENOME Human p62 siRNA SMARTpool (M-010230-00-005) and siGENOME Non-Target siRNA pool#1 (D-001206-13-05) were purchased from Horizon Discovery Biosciences Limited (Cambridge, UK). The transfection was performed, as described previously (17). p62 knockdown was assessed by immunoblotting.

### MTT and Clonogenic Assays

MTT assay was performed as described previously (18). Colony formation was performed as described elsewhere (19). IC50 values were obtained using the GraphPad Prism software. CalcuSyn drug dose-effect analysis software (CalcuSyn V2, Biosoft, Cambridge, UK) was used to determine synergy (combination indexes and isobolograms).

### Immunoblot Analysis

Whole-cell lysate preparation and immunoblot were performed, as described previously (18, 20).

### Comet Assay

Twenty thousand cells were used for single-cell gel electrophoresis using the Trevigen comet assay kit (Trevigen Inc., Gaithersburg, MD). Neutral and modified alkaline assays were performed as described previously (21–23) with slight modification. The neutral and alkaline comets were stained with propidium iodide and SYBR Gold, respectively. A total of 50 images were obtained per group under the Nikon Ti-DH fluorescent microscope. Images were analyzed using ImageJ (NIH, USA).

### Proteomic Analysis

SUM159-P and SUM159-R cells were harvested and stored at -80°C. A total of 5 biological replicates for parental and olaparib-resistant cells each were coded and submitted to the Wayne State University Proteomic Core for further processing. For global proteomic analysis, pellets from isolated cells were reduced/alkylated with DTT/IAA before digestion with trypsin. Digestion was evaluated by running an aliquot of each sample through SDS-PAGE as a “Before” and “After” addition of trypsin. Each digest was labeled with TMT-11plx reagents, and the individual samples were evaluated by mass spectrometry for labeling completeness. Once greater than 99% labeling was confirmed, five samples were pooled and fractionated into 9 fractions by alkaline reversed-phase spin column. The fractions were analyzed by LC-MS/MS on an orbitrap Fusion MS system. All data were analyzed using Proteome Discoverer and MaxQuant software.

### Statistical Analysis

All data were analyzed with R, GraphPad Prism, or Microsoft Excel. Data were presented as mean ± standard deviation (SD). Comparisons between groups were made using Student’s t-test. Kaplan-Meier curves were used to graphically summarize overall survival. A log-rank test was used to compare between two survival curves and a hazard ratio (HR) was estimated using Cox proportional hazard models. All survival analyses were performed using the webtool Kaplan-Meier Plotter (http://kmplot.com) (24).

## Results

### Olaparib-Resistant TNBC Cells Display Impaired DNA Damage Responses

**Figure 1:**
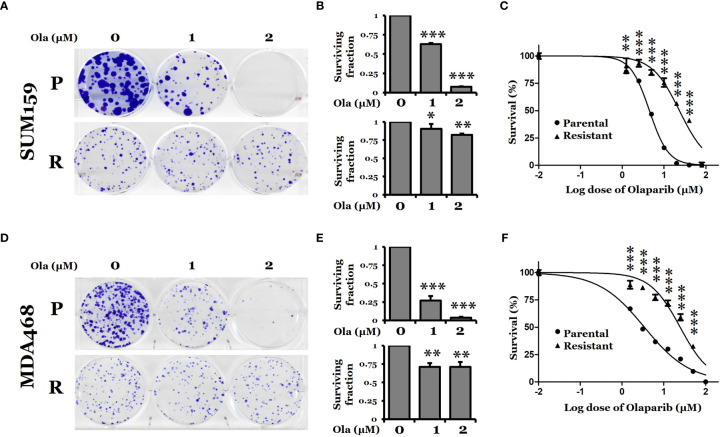
Clonogenic survival of PARP inhibitor olaparib-sensitive and -resistant TNBC cells. **(A, D)** Colony formation assay for SUM159 and MDA468 parental (P) and olaparib resistant (R) cells. Cells were treated with either DMSO or olaparib for 72 hrs and allowed to grow in a drug-free medium for 10 days. All treatments were carried out in triplicate, and the images are representative of the response to olaparib. **(B, E)** Surviving fraction calculated from A and D, respectively. **(C, F)** Dose-response curve for parental and olaparib resistant SUM159 and MDA468 cells. Resistant cells were obtained by selection with increasing concentrations of olaparib in the culture media for six months. After the development of resistance, SUM159-R and MDA468-R cells were maintained in media containing 25 µM and 15 µM olaparib, respectively. Cell viability was measured using MTT assay after treating with varying concentrations of olaparib for 72 hrs. After adding MTT to the media, cells were incubated at 37˚C for 2 hrs. The formazan crystals were dissolved in DMSO and then read by spectrophotometer at 570 nm. Data represented as mean ± standard deviation (SD). All experiments were done in triplicates. Ola, olaparib. Student’s t-test: *p <0.05; **P <0.01 and ***P <0.001.

To gain insights into the mechanism of PARPi resistance, we established two PARPi-resistant cell lines, SUM159-R and MDA468-R, by exposing parental SUM159 (SUM159-P) and MDA468 (MDA468-P) cells to increasing concentrations of olaparib over six months. Both SUM159 and MDA468 cells are sensitive to olaparib and have intact BRCA genes, while MDA468 cells are HR defective (25). We chose these two lines because we were interested in the mechanism by which wild-type (wt) BRCA1/2, PARPi-sensitive TNBC cells develop acquired PARPi resistance. We found that resistant cells proliferated slightly slower than corresponding parental cells (data not shown) and that olaparib significantly inhibits colony formation in SUM159-P and MDA468-P cells compared to their corresponding SUM159-R and MDA468-R cells (**Figures 1A, B, D, E**). **Figure 1C** shows that IC50s of SUM159-R and SUM159-P cells were 23.3 μM and 4.5 μM, respectively. Similarly, IC50s of MDA468-R cells and MDA468-P were 24.6 µM and 3.4 µM, respectively (**Figure 1F**).

**Figure 2:**
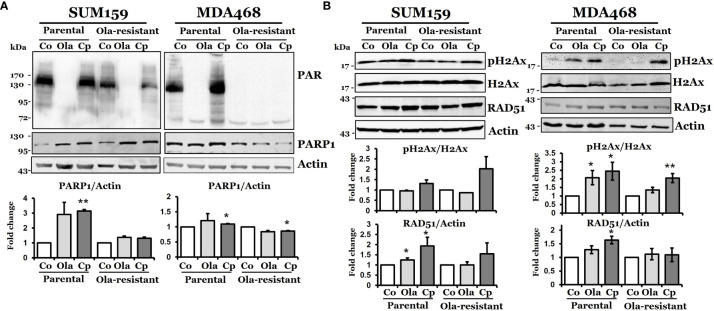
DNA damage response in olaparib-sensitive and -resistant TNBC cells. Western blot analysis of DNA damage response associated proteins, PAR, PARP-1 **(A)**, pH2AX, and RAD51 **(B)** in parental and olaparib resistant SUM159 and MDA468 cells. Cells were treated with 5 µM olaparib or 2 µM cisplatin for 24 hrs. SUM159-R and MDA468-R cells were maintained in drug-free media 2-3 days before the experiment. For the determination of band density, NIH ImageJ 1.5Oi software was used. Band densities were normalized against Actin or total H2AX. Images are representative of three independent experiments. Co, control; Ola, olaparib and Cp, cisplatin. Student’s t-test: *p < 0.05 and **p < 0.01.

Next, we characterized DNA damage responses with olaparib or cisplatin treatment in these olaparib-resistant cells. We first measured the expression of PARP1. **Figure 2A** shows that the basal and cisplatin- and olaparib-induced PARP1 levels were similar between SUM159-P and SUM159-R cells. The induction of PARP1 in SUM159-P cells under cisplatin treatment was statistically significant (*p* = 0.002). PARP1 levels were increased by cisplatin in MDA468-P cells (*p* = 0.022) but decreased in MDA468-R cells (*p* = 0.019). We then showed that poly (ADP-ribose) (PAR) levels were significantly and completely inhibited in untreated SUM159-R cells and MDA468-R cells, respectively, compared to untreated parental cells. While olaparib inhibited PAR levels in both SUM159-P and MDA468-P cells, cisplatin slightly increased PAR levels in SUM159-P and MDA468-P cells. However, PAR levels were slightly decreased and remained undetectable in cisplatin-treated SUM159-R and MDA468-R cells, respectively (**Figure 2A**). These data suggest that basal and induced PAR activity are significantly inhibited in olaparib-resistant cells. We also found that γ-H2AX levels were modestly elevated in SUM159-P and SUM159-R cells by cisplatin but remained unaffected by olaparib (**Figure 2B**). While the basal level of γ-H2AX was lower in MDA468-R cells over MDA468-P cells, either olaparib (*p* = 0.041) or cisplatin (*p* = 0.031) significantly increased γ-H2AX in MDA468-P cells, which was only observed by cisplatin (*p* = 0.007) but not by olaparib (*p* = 0.079) in MDA468-R cells (**Figure 2B**). Furthermore, we found that RAD51 levels were induced by olaparib or cisplatin in SUM159-P and MDA468-P cells, while a modest increase in RAD51 level was detected in cisplatin, not olaparib treated SUM159-R cells. By contrast, neither olaparib nor cisplatin significantly increased RAD51 levels in MDA468-R cells (**Figure 2B**). These data suggest that PARP enzymatic activity is significantly inhibited in olaparib-resistant cells.

### Olaparib-Resistant TNBC Cells Exhibit Different Repair Capacities in Response to Cisplatin and Olaparib

**Figure 3:**
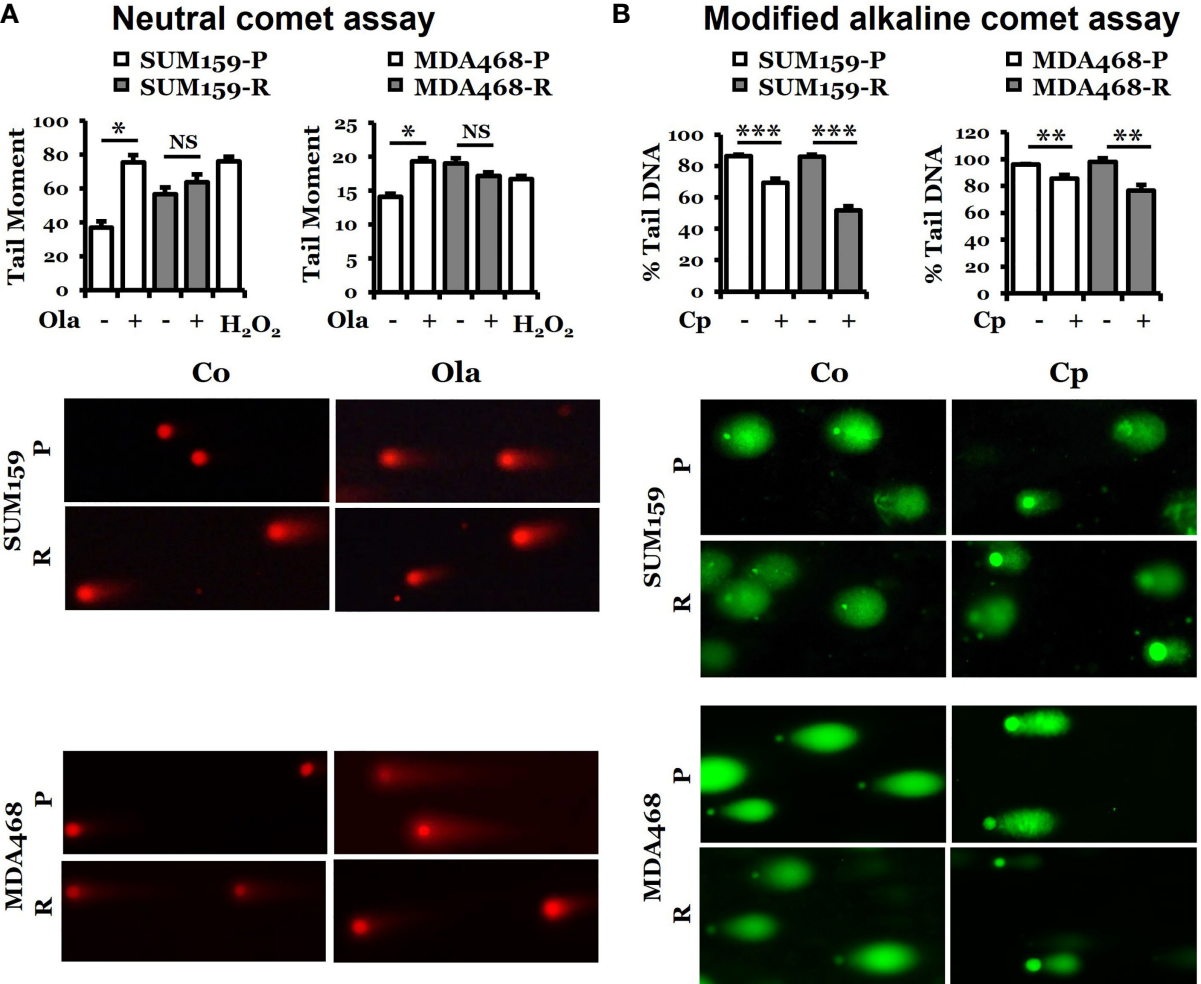
Determination of DNA damage response in parental and olaparib resistant SUM159 and MDA468 cells by comet assays. **(A)** Neutral comet assay for detecting double-strand breaks in DNA in the form of tail moment. Parental and olaparib resistant SUM159 and MDA468 cells were treated with 15 µM and 10 µM olaparib for 24 hrs, respectively, followed by the electrophoresis of nuclear DNA. An average of 50 cells was used to calculate the tail moments. The lower panel shows a representative image of each group. **(B)** Modified alkaline comet assay for detecting DNA crosslinks in the form of percent tail DNA. Parental and olaparib resistant SUM159 and MDA468 cells were treated with 2 µM cisplatin for 24 hrs before the electrophoresis of nuclear DNA. An average of 50 cells was used to calculate the percent tail DNA. The lower panel shows a representative image from each group. Co, control; Ola, olaparib; Cp, cisplatin; P, parental; R, resistant and NS, not significant. Student’s t-test: *p < 0.05; **p < 0.01 and ***p < 0.001.

Since olaparib can cause double-strand breaks (26), we performed a neutral comet assay for the tail moment to assess the capacity of DNA damage response in both olaparib-sensitive and -resistant cells. **Figure 3A** shows that the tail moment significantly increased in both SUM159-P and MDA468-P cells, but such changes were not detected in SUM159-R and MDA468-R cells. Interestingly, there was a higher level of basal double-strand breaks (DSBs) in olaparib-resistant cells, but this level did not change with olaparib treatment. Since the adaptive olaparib resistant cells were derived by continuous exposure to olaparib, the high levels of DNA damage experienced by these cells result from olaparib-induced stress, which are not further increased by olaparib indicating saturation levels of DNA damage in these cells. We then used another DNA damaging agent, cisplatin, to detect DNA tail formation because cisplatin is known to cause DNA crosslinking. **Figure 3B** shows that cisplatin reduced tail DNA significantly in both parental and olaparib-resistant cell lines. These data suggest that olaparib-resistant cells can respond to cisplatin-induced DNA damage, but such capacity was decreased in responding to olaparib treatment.

### p62 Is a Critical Player in Olaparib Resistance in TNBC Cells

**Figure 4:**
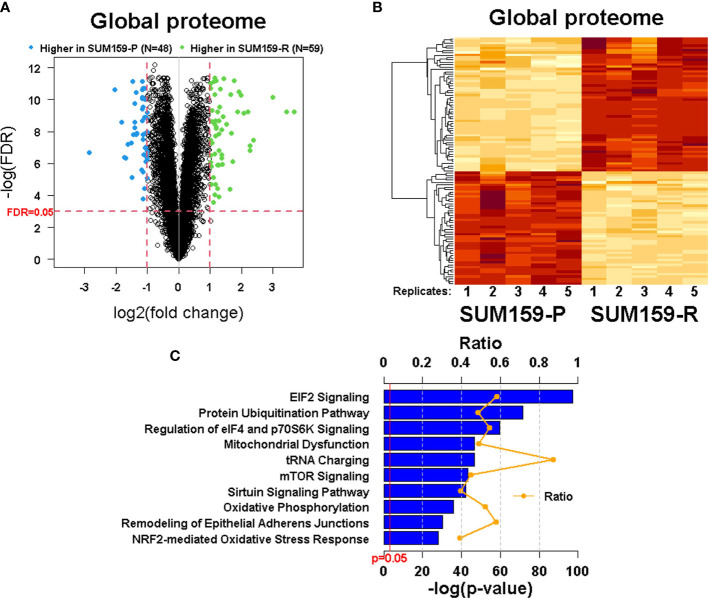
Quantitative measurement of the global proteome using TMT labeling in SUM159 parental and resistant TNBC cells. **(A)** Volcano plots showing differentially expressed proteins in SUM159-R cells compared to SUM159-P cells. Each dot represents one protein. Blue and green dots represent proteins that are significantly higher in SUM159-P cells (N = 48) and those that are significantly higher in SUM159-R cells (N = 59), respectively, with FDR<0.05 and fold change (FC) ≥ 2. **(B)** Heatmap generated from proteins detected in all samples by hierarchical clustering. FDR<0.05 and FC ≥2. **(C)** Top 10 canonical pathways related to olaparib resistance derived from the global proteome’s ingenuity pathway analysis (IPA). P, parental and R, resistant.

To understand the mechanism of acquired olaparib resistance in these TNBC cells, we performed a global proteomic analysis using TMT-labeling, and high-resolution LC-MS/MS followed by differential expression analysis in SUM159-P and SUM159-R cells. From the five biological replicates of the global proteomic profiles, we identified 6 447 proteins and, of these, 107 proteins were differentially expressed between SUM159-P and SUM159-R cells with FDR<0.05 and fold change ≥ 2 (**Figure 4A**). Clustering of the data (Heatmap) showed their distinct patterns between olaparib-sensitive and -resistant cells (**Figure 4B**). Ingenuity pathway analysis (IPA) identified a number of canonical pathways significantly altered between SUM159-P and SUM159-R cells, including the EIF2, mTOR, ubiquitination, DNA repair, and mitochondrial pathways (**Figure 4C**).

**Figure 5:**
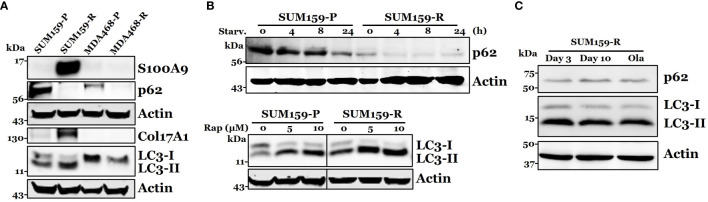
Confirmation of S100A9, p62, COL17A1, and LC3 expression in olaparib resistant cells. **(A)** Western blot analysis of S100A9, p62, COL17A1, and p62 associated protein LC3-I, II in both parental and resistant SUM159 and MDA468 cells. **(B)** Effect of autophagy stimulation on p62 and LC3 expression. Parental and resistant SUM159 cells were starved for indicated periods (upper panel) or treated with two different doses (5 µM and 10 µM) of rapamycin for 24 hrs (lower panel). The levels of p62 or LC3 were measured by western blotting. Rap, rapamycin and starv, starvation. **(C)** Effect of olaparib withdrawal on LC3 and p62 levels. SUM159-R cells were maintained in olaparib-free media for 3 days and 10 days or in olaparib-containing (25 µM) medium (ola).). P, parental; R, resistant; Starv., starvation; Rap, rapamycin; and Ola, olaparib.

Among top differentially expressed proteins, we identified several proteins that could be associated with olaparib resistance, including S100A9, p62, and COL17A1. We performed immunoblot analysis to verify the expression of S100A9, p62, and COL17A1. We found that p62 was down-regulated in both SUM159-R and MDA468-R cells compared to their parental cells, while S100A9 and COL17A overexpression were only confirmed in SUM159-R but not in MDA468-R cells (**Figure 5A**). Because decreased p62 expression was detected in both olaparib-resistant lines, we focused our study on the role of p62 in olaparib resistance. Since p62 plays a critical role in autophagy, we asked if lower p62 levels in olaparib-resistant cells are due to higher autophagic activity. To test this possibility, we assessed LC3 conversion and showed that decreased LC3-I levels in SUM159-R cells accompanied increased LC3-II levels compared to SUM159-P cells (**Figure 5B**). Although LC3-II levels were low in both olaparib-sensitive and -resistant cells, LC3-I levels were significantly lower in MDA468-R than MDA468-P cells (**Figure 5A**), suggesting that lower LC3-I levels in MDA468-R cells may be due to increased conversion of LC3-I to LC3-II. Next, we asked if olaparib-resistant cells can still respond to external autophagy stimulation. We starved SUM159 cells and showed that starvation of SUM159-P and SUM159-R cells gradually caused a decrease in p62 levels (**Figure 5B**), although SUM159-P and SUM159-R cells expressed different steady-state levels of p62. We then treated these cells with rapamycin to induce autophagy and showed that rapamycin treatment increased LC3-II levels (**Figure 5B** lower panel). Furthermore, we showed that SUM159-R cells maintained high LC3-II and lower p62 levels when these cells were maintained in olaparib-free media (**Figure 5C**). These data suggest that although SUM159-R cells have high steady-state levels of autophagy compared to SUM159-P cells, SUM159-R cells can still elicit heightened external autophagy responses.

### mTOR Inhibitors Synergistically Sensitize Olaparib-Resistant TNBC Cells to Olaparib

**Figure 6:**
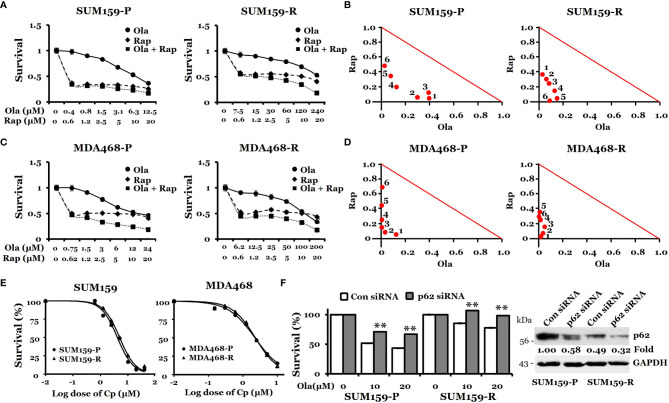
Effects of rapamycin plus olaparib or cisplatin alone on the growth of olaparib-resistant TNBC cells. **(A, C)**, Synergistic effects of rapamycin and olaparib treatment on the growth of SUM159-P and SUM159-R cells **(A)** and MDA468-P and MDA468-R cells **(C)**. Cells were treated with indicated drug combination for 72 hrs. **(B, D)**, Normalized isobologram for SUM159-P and SUM159-R cells **(B)** and MDA468-P and MDA468-R cells **(D)**. **(E)** Sensitivity of olaparib-resistant cells to cisplatin in parental and olaparib resistant SUM159 cells (left) and MDA468 cells (right). Cell viability was measured using MTT assay after treatment with varying concentrations of cisplatin for 72 hrs. All experiments were performed in triplicates. P, parental; R, resistant; Ola, olaparib; Cp, cisplatin; Rap, rapamycin and con, control. **(F)** Effect of p62 knockdown on olaparib sensitivity in SUM159 cells. SUM159-P and SUM159-R cells were transfected with p62 siRNA or non-target siRNA (Con siRNA). MTT was performed to assess olaparib sensitivity (left panel), and the level of p62 knockdown was evaluated by western blotting (right panel). Student’s t-test: **p < 0.01.

Next, we asked if further promoting/hyper-activating autophagy in these olaparib-resistant cells can be therapeutically exploited for overcoming olaparib resistance. To this end, both parental and olaparib-resistant SUM159 and MDA468 cells were treated with olaparib, rapamycin, or combination. We found that rapamycin alone has a modest effect against both parental and olaparib-resistant cells. Importantly, the combination of rapamycin with olaparib significantly inhibited the growth of both parental and olaparib-resistant SUM159 and MDA468 cells (**Figures 6A, C**), and such effects were synergistic (combination index (CI) <1), which was evident from normalized isobologram (**Figures 6B, D**). Specifically, the CI values were as low as 0.078 and 0.053 for SUM159-R and MDA468-R cells, respectively, indicating strong synergistic effects when combining olaparib and rapamycin. Synergy was also observed in parental SUM159 (CI = 0.328 – 0.513) and MDA468 (CI = 0.129 – 0.663) cells. Similar results were obtained with another mTOR inhibitor, everolimus (**Figure S1**). We also found that p62 knockdown renders both SUM159-P and SUM159-R cells more resistant to olaparib than corresponding cells transfected with non-target siRNA (**Figure 6F**). These data suggest that p62 may play a general role in olaparib sensitivity and that promoting autophagy is likely responsible for synergistic effects by olaparib and mTOR inhibitors. Interestingly, we found that the autophagy inhibitor chloroquine enhances olaparib-induced cell death (**Figure S2**), suggesting that either enhancing or inhibiting autophagy may sensitize olaparib-resistant cells to olaparib.

Since SUM159-R cells slightly increased PAR levels by cisplatin (**Figure 2**), we suspected that these olaparib-resistant cells are sensitive to cisplatin. Accordingly, we treated both parental- and olaparib-resistant cells with cisplatin and assessed growth inhibition. IC50 for cisplatin was 4.1 µM and 4.9 µM in SUM159-P and SUM159-R cells, respectively (**Figure 6E**). IC50 for cisplatin was 2.2 µM and 2.3 µM in MDA468-P and MDA468-R cells, respectively (**Figure 6E**). Taken together, our data suggest that olaparib-resistant TNBC cells can be effectively treated by cisplatin or PARPi plus rapamycin.

### p62 Protein Expression Is Correlated With Better Overall Survival in Estrogen Receptor (ER) Negative Breast Cancer

**Figure 7:**
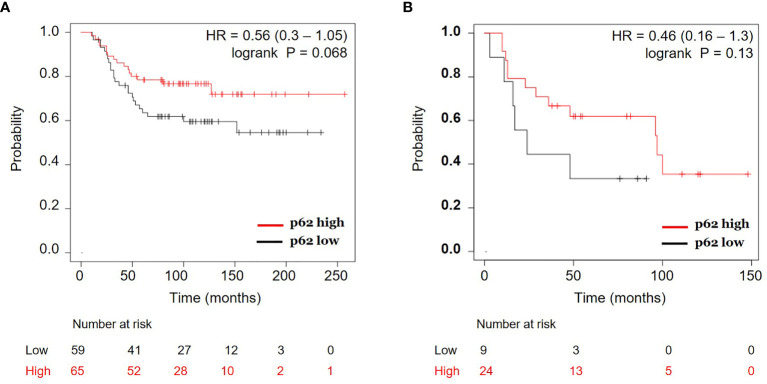
Correlation of p62 protein expression and overall survival in estrogen receptor (ER) negative breast cancer. Kaplan-Meier curves of ER-negative breast cancer patients with p62 protein high vs. p62 protein low for overall survival were generated using the webtool Kaplan-Meier Plotter (http://kmplot.com) (24) using two breast cancer datasets: **(A)** Liu et al. (2014) (27) and **(B)** Tang et al. (2018) (28). HR, hazard ratio.

To address the clinical relevance of p62 expression, we analyzed p62 protein expression versus patient survival in the breast cancer dataset from Liu et al. (2014) (27). **Figure 7A** shows that ER-negative patients with higher p62 protein levels have better overall survival than those with lower p62 protein in 124 patients (HR, 0.56; 95% CI, 0.3 to 1.05; *p* = 0.068). Consistently, further analysis of another** **breast cancer dataset from Tang et al. (2018) (28) revealed a similar positive correlation between overall survival and p62 protein expression in 33 ER-negative breast cancer patients (HR, 0.46; 95% CI, 0.16 to 1.3; *p* = 0.13) (**Figure 7B**). Although the number of patients for each group is low and low p62 protein level is not absolutely an indication of high autophagy, and considering a critical role p62 plays during autophagy, our proof-of-principle data suggest that high autophagic activity (low p62 levels) is correlated with poor survival in ER-negative breast cancer patients and those patients may benefit from cisplatin or PARPi plus rapamycin treatments.

## Discussion

PARPis are increasingly being used to treat human cancers, particularly for tumors with BRCA1/2 mutations or BRCAness; however, the development of acquired resistance limits their use in the clinic. In this study, we generated PARPi-resistant TNBC cells and analyzed the global proteomic profiling for altered signaling pathways that may be responsible for the development of PARPi resistance. We identified a number of pathways associated with PARPi resistance, focusing on autophagy-associated protein p62 in olaparib resistance in TNBC cells.

p62 plays a critical role in autophagy (29), and a reduction of p62 levels is a consequence of enhanced autophagy associated with enhanced cell survival (30). We found that p62 protein levels were decreased in olaparib-resistant TNBC cells. Our observation of decreased p62 levels indicates that these olaparib-resistant cells have hyperactivated levels of autophagy compared to their olaparib-sensitive counterparts. Consistently, we found that LC3-I and LC3-II levels were higher and lower in olaparib-sensitive cells, respectively, and vice versa in olaparib-resistant TNBC cells. Since decreased LC3-I/increased LC3-II and decreased p62 levels are hallmarks of autophagy, our data suggest that TNBC cells gain autophagic activity as these olaparib-sensitive cells develop olaparib resistance.

Previous studies identified PARPi-induced autophagy as a mechanism of PARPi resistance (31, 32). In these studies, acute PARPi treatment activated the autophagy pathway, and inhibiting autophagy enhanced PARPi sensitivity in breast and ovarian cancer cells (31, 32). These studies showed autophagy activation as a resistance mechanism. In contrast, this study used an unbiased approach, namely LC-MS/MS, to identify differentially expressed proteins between olaparib-sensitive vs. olaparib-resistant clones, which led to the identification of p62 loss in olaparib-resistant cells. Importantly, we showed that autophagy-promoting agents could effectively inhibit the growth of PARPi-resistant TNBC cells with higher autophagic activity. Interestingly, we showed that the autophagy inhibitor chloroquine enhances olaparib-induced cell death (**Figure S2**), which seems inconsistent with the data obtained from rapamycin, everolimus, and p62 knockdown experiments. This suggests that in some cells (e.g., SUM159 cells) with high autophagic activity, either enhancing autophagy or inhibiting autophagy may sensitize olaparib-resistant cells to olaparib and this possibility is under investigation. Although chloroquine can inhibit autophagy, enhancing olaparib sensitivity by chloroquine could be due to its non-autophagy, general growth inhibitory effect. On the other hand, rapamycin and everolimus-mediated growth inhibition could be through an autophagy-independent mechanism. It is noteworthy that knockdown of p62 decreases olaparib sensitivity in SUM159-P and SUM159-R cells, suggesting the role of p62-mediated autophagy in olaparib sensitivity.

It has been demonstrated that mTOR signaling can be upregulated by cisplatin in *in vivo* models, which is associated with the degradation of p62 (33). Upregulation of mTOR signaling in olaparib-resistant cells may lead to the loss of p62 *via* some currently not understood mechanisms. Rapamycin treatment may reverse this process by downregulating mTOR signaling. However, based on the data obtained from rapamycin, everolimus, and p62 knockdown experiments, we argued that the inhibitory effect of rapamycin/everolimus on the growth of olaparib-resistant cells is partially *via* induction of autophagy. Considering the effects of rapamycin/everolimus on mTOR and several other signaling pathways, we believed that in addition to the impact on autophagy induction, the synergistic effects of PARPi and mTOR inhibitors on the growth of these TNBC cells can be attributable to inhibiting cell survival signaling pathways and promoting programmed cell death.

Our pathway analysis identified a number of pathways that were significantly altered between SUM159-P and SUM159-R cells, including elF4 and mTOR (**Figure 4**). It has been known that eukaryotic initiation factors can regulate cell survival and that the inhibition of mTOR and elF4 can promote cell death. Consistently, phosphorylation of eIF2α by mTORC1 inhibition is required for autophagy (34). Because there are connections between PARP-1-mediated autophagy, the AMPK/mTOR pathway, and AMPK-regulated cell growth (35, 36), it is interesting to investigate if AMPK plays a role in olaparib resistance in those olaparib-resistant TNBC cells.

We showed that olaparib-resistant cells display impaired DNA damage response and repair pathways (**Figure 2**). This is not surprising since such defects in DNA damage response/repair pathways have been implicated in PARPi resistance in several types of cancer (37). While the contributions of DNA damage/repair pathways to PARPi resistance are currently being intensively studied, we wanted to identify non-DNA repair mechanisms that may play roles in PARPi resistance. Our study supports the role of p62-mediated autophagy in olaparib resistance in TNBC cells. Since PARPi resistance mechanisms can be multifactorial, including restoration of the BRCA gene by acquiring secondary mutations, identifying p62-mediated PARPi resistance likely augments other resistance mechanisms for rationally designing better approaches to overcome PARPi resistance.

Loss of p62 has been observed in multiple types of cancer (38). For example, p62 loss in tumor-adjacent stromal cells resulted in reprogramming the tumor microenvironment that favors tumor growth and that p62 loss in adipocytes and stromal fibroblast promotes tumorigenesis (39, 40). Consistently, we found that decreased p62 expression is negatively correlated with survival in ER-negative breast cancer patients. Considering the critical role p62 plays in autophagy, these ER-negative breast cancers may have high autophagy and that enhanced autophagy may negatively impact the survival of these patients. Therapeutically, on the other hand, these patients may benefit from the combination of olaparib and rapamycin treatment. While p62 protein expression was correlated with better overall survival in ER-negative breast cancer (**Figure 7**), it is believed that accumulating p62 can be detrimental to some cells in response to stress (30, 41). Therefore, the role of p62 in cancer is perplexing, which is in part dependent on cell types and stimuli.

In conclusion, we found that p62 plays a crucial role in olaparib resistance in TNBC cells. Consequently, targeting p62-mediated autophagy by hyper-activating autophagy can sensitize olaparib-resistant TNBC cells to PARPi. Therefore, our data suggest that targeting autophagy could improve outcomes for TNBC patients who have undergone PARPi-based treatments but developed PARPi resistance.

## Data Availability Statement

The datasets presented in this study can be found in online repositories. The names of the repository/repositories and accession number(s) can be found below: https://figshare.com/, http://dx.doi.org/10.6084/m9.figshare.17204099.

## Author Contributions

GW and MS conceived, MS designed, analyzed the results, and coordinated the project. M-HU, J-YZ, and JP conducted the experiments and analyzed the data. SP assisted with Comet assay. SK assisted with RNA-seq and database analysis. M-HU and GW wrote the original draft. GW, M-HU, MS, J-YZ, JP, SP, and SK reviewed and edited. All authors contributed to the article and approved the submitted version.

## Funding

This work was, in part, supported by National Institutes of Health Grant R01CA174949, 1R21CA249376 and DMC Foundation (GW), T32-CA009531 (JP), R01CA229535 (SP), and a Strategic Research Initiative grant from Karmanos Cancer Institute (to MS and GW).

## Conflict of Interest

The authors declare that the research was conducted in the absence of any commercial or financial relationships that could be construed as a potential conflict of interest.

## Publisher’s Note

All claims expressed in this article are solely those of the authors and do not necessarily represent those of their affiliated organizations, or those of the publisher, the editors and the reviewers. Any product that may be evaluated in this article, or claim that may be made by its manufacturer, is not guaranteed or endorsed by the publisher.

## Supplementary Material

Supplementary Figure 1 Effects of everolimus in combination with olaparib in the growth of olaparib-sensitive and olaparib-resistant SUM159 cells. A and B, Synergistic effects of everolimus and olaparib treatment on the growth of SUM159-P and SUM159-R cells respectively. Cells were treated with indicated drug combination for 72 hrs. Middle table, combination index (CI) values. Middle panel, graphical presentation of combination index along with the affected fraction (Fa). Right panel, normalized isobologram. Ola, olaparib; Eva, evarolimus.

Supplementary Figure 2 Effects of chloroquine in combination with olaparib in the growth of olaparib-sensitive and olaparib-resistant SUM159 cells. A and B, Synergistic effects of chloroquine and olaparib treatment on the growth of SUM159-P and SUM159-R cells respectively. Cells were treated with indicated drug combination for 72 hrs. Middle table, combination index (CI) values. Middle panel, graphical presentation of combination index along with the affected fraction (Fa). Right panel, normalized isobologram. Ola, olaparib; Chq, chloroquine.
