# Supplementary figures and images for: Proteomic Analysis Identifies p62/SQSTM1 as a Critical Player in PARP Inhibitor Resistance
Source: Front Oncol. 2022 Jun 29;12:908603. doi: 10.3389/fonc.2022.908603 (PMC9277186; doi:10.3389/fonc.2022.908603)

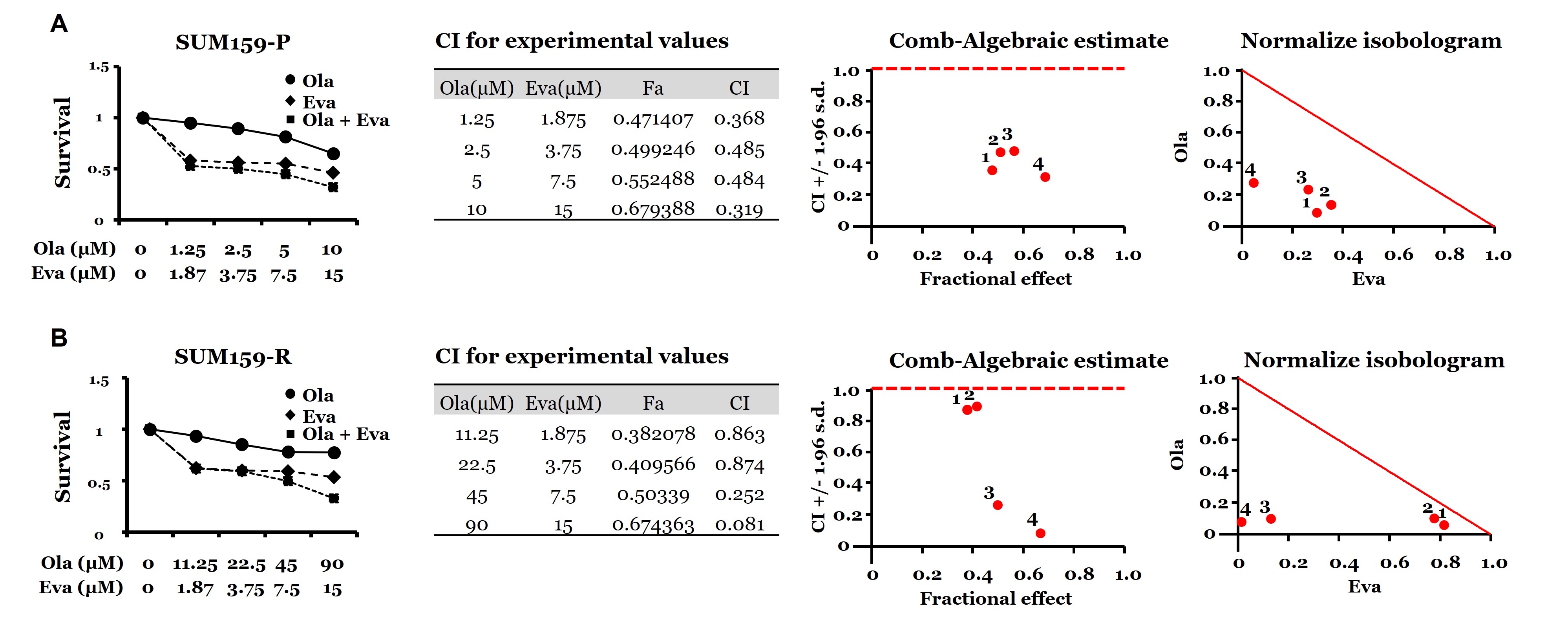

Supplement: Supplementary Figure 1 — Effects of everolimus in combination with olaparib in the growth of olaparib-sensitive and olaparib-resistant SUM159 cells. A and B, Synergistic effects of everolimus and olaparib treatment on the growth of SUM159-P and SUM159-R cells respectively. Cells were treated with indicated drug combination for 72 hrs. Middle table, combination index (CI) values. Middle panel, graphical presentation of combination index along with the affected fraction (Fa). Right panel, normalized isobologram. Ola, olaparib; Eva, evarolimus. [file Image_1.jpg]

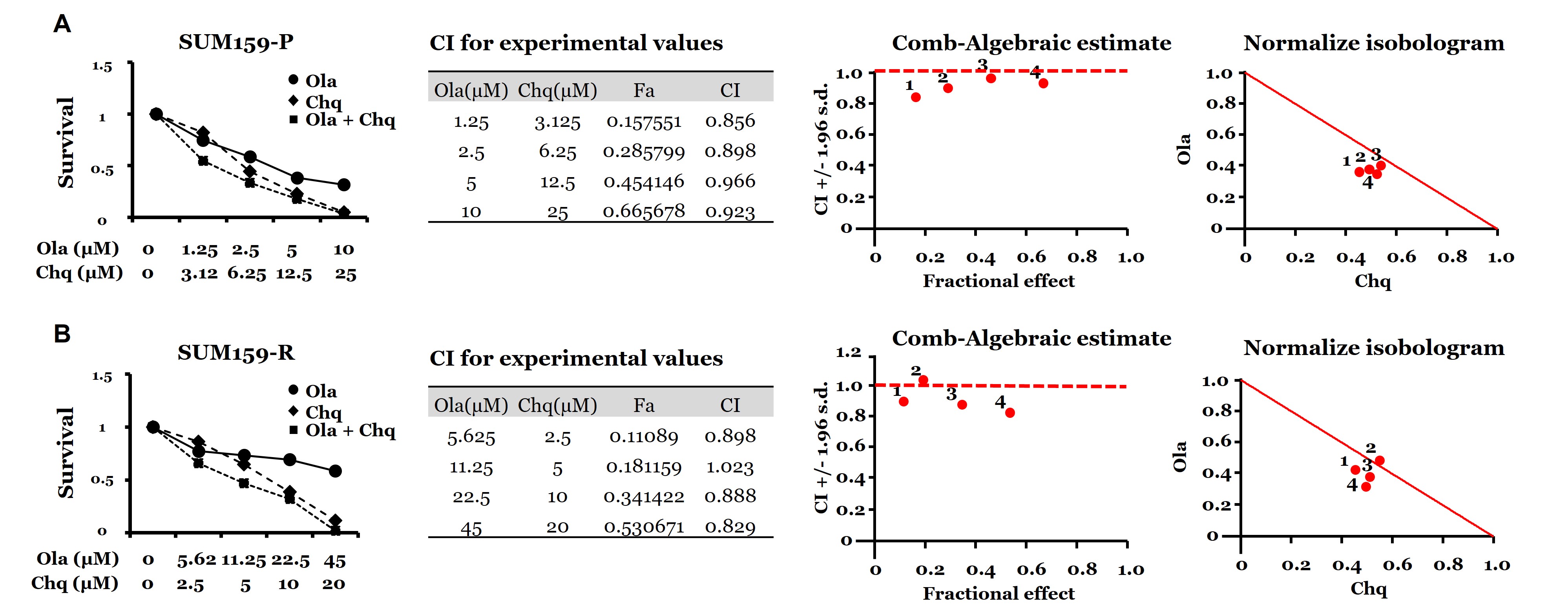

Supplement: Supplementary Figure 2 — Effects of chloroquine in combination with olaparib in the growth of olaparib-sensitive and olaparib-resistant SUM159 cells. A and B, Synergistic effects of chloroquine and olaparib treatment on the growth of SUM159-P and SUM159-R cells respectively. Cells were treated with indicated drug combination for 72 hrs. Middle table, combination index (CI) values. Middle panel, graphical presentation of combination index along with the affected fraction (Fa). Right panel, normalized isobologram. Ola, olaparib; Chq, chloroquine. [file Image_2.jpg]
